# Biofungicides for Improvement of Potato (*Solanum tuberosum* L) Production

**DOI:** 10.1155/2022/1405900

**Published:** 2022-10-03

**Authors:** John Ehiobu, Emrobowansan Idamokoro, Anthony Afolayan

**Affiliations:** ^1^Medicinal Plants and Economic Development (MPED) Research Centre, Botany Department, University of Fort Hare, Alice 5700, South Africa; ^2^Faculty of Commerce and Administration, Department of Economics and Business Science, Walter Sisulu University, P/Bag X1, Mthatha 5117, South Africa

## Abstract

Potato (*Solanum tuberosum* L) cultivation originated from Peru in Latin America. The cultivation has spread fast across the globe due to its ability to cope in the warm tropical and temperate climate. It is spotted by the United Nations as the only tuberous crop that can compete with the cereals in productivity. Fungal disease infestation has been identified as a major challenge confronting the farmers during the cultivation and marketing processes. Farmers' reliance on Chemical fungicides has lost its credibility to the adoption of the use of biofungicides due to its toxic, high cost, and environmental hazard effects. The trend of the adoption of biofungicides by potato farmers is gaining ground at a fast rate. Various national governments are devising means of collaborating with the United Nations stakeholders through encouraging research funding and by organizing conferences that will enhance potato production. This could be achieved by minimizing losses through farmer's complete adoption of biofungicides. This review, therefore, examines the various botanicals with antimicrobial properties as potential biofungicide against fungi diseases of potato.

## 1. Introduction

Potato cultivation history is traced in a country called Peru in Latin America between 8000BC-5000BC [1]. In the year 1556, Spanish conquistadors conquered Peru and discovered the desirable taste and flavor of potato that resulted in its introduction in Europe. Sir Walter Raleigh introduced potato to Ireland in the year 1589 on 40,000 hectares of land near Cork. History further disclosed that it took nearly four decades for the consumption and cultivation of potato to spread to the rest of Europe. The less intensive labor involved in the growth and cultivation of potato compared to other crops such as wheat, oats and the rich nutrient content led to the fast adoption of the crop by farmers in Europe. Furthermore, potato was introduced by the governor of Bermuda, called Nathaniel Butler to America in 1621. The introduction of potato further spread to North America in 1719. It was reported that potato was introduced in South Africa by a Dutch seaman that was sailing towards Asia in the 1600s. In October 1995, potato became the first vegetable to be grown in Space. National Aeronautics and Space Administration (NASA) in collaboration with the University of Wisconsin embarked on the project with the objective of feeding astronauts on long space voyages. South Africa has been identified as one of the greatest producers of potato in Africa. Today potato is the fourth food crop extensively cultivated globally. A large amount of food from potato is not reaching consumers due to postharvest losses [2].

The global consumption and the growth of *Solanum tuberosum* L. are on the increase despite the loss constraint from fungal pathogens infection at both pre- and post-harvest stages of production [3]. Chemical fungicides have been used for controlling fungal causing diseases in crop plants generally, including potato rots, scabs, and blight diseases [4]. With time, chemical fungicide usage has increasingly become unpopular due to the awareness of the high cost, toxicity, and polluting effects [5]. This has necessitated the need for alternative sources of fungicides that can be developed from plants. Several works have been reported on antifungal activities of plants extracts against fungi pathogens of wheat, rice, pea, tomato, and other relevant crops [6–8]. This paper examines plants with antimicrobial potentials as biofungicides for the control of potato diseases.

## 2. The Botanical Description of *Solanum tuberosum*

**Figure 1:**
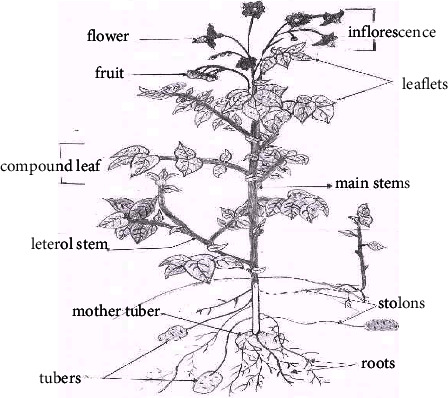
An annotated diagrammatic presentation of a matured potato plant (*Solanum tuberosum* L) [9].

*Solanum tuberosum* L. is an herbaceous perennial plant in the family of Solanaceae and it is grown for its edible tubers [9]. The matured potato plant has a branched stem and alternately arranged leaves, consisting of leaflets that are both of unequal size and shape. The leaflets can be oval to oblong in shape and the leaves size is about 10–30 cm in length and 5–15 cm wide. The matured potato plant produces white or purple and yellowish green berry fruits. The potato tubers grow underground and are generally located on the top 25 cm of the soil. The tuber color varies from red, purple, and yellow depending on the variety. A matured potato plant is about 1 meter in height and are grown as annuals that survive only one growing season [9].  Figure 1 shows the diagram of a matured potato plant (*Solanum tuberosum* L).

Below is the Linnaeus hierarchical system of classification:

 
*Kingdom: Plantae* (Plants)
 
*Subkingdom*: Tracheobionta (Vascular plants)
 
*Super division*: Spermatophyta (Seed plant)
 
*Division*: Magnoliophyta (Flowering plants)
 
*Class*: Magnoliopsida (Dicotyledon)
 
*Subclass*: Asteridae
 
*Order*: Solanales
 
*Family*: Solanaceae
 
*Genus*: *Solanum* L
 
*Species*: *Solanum tuberosum* L [10].

## 3. Nutrient Composition of *Solanum tuberosum*

Potato tubers consist of macronutrients such as water, indigestible carbohydrates, and other health-beneficial ingredients such as protein and fibers and micronutrients such as vitamins C, potassium, magnesium, and phytonutrients in form of carotenoid and phenolic acid [11]. The raw potato must be processed before human consumption to make starch and other nutrients bioavailable. The nutritional value of potato along with the taste and ease of cooking has made it the most popular vegetable and snack in the World [12]. During the winter season in Europe, a potato is mostly relied upon for food supply [13]. In addition to the contents of potato mentioned above, it has also been reported to contain a lot of phytochemicals that are antioxidants. Antioxidant compounds are compounds that stop or reduce the oxidative processes and consequently delay or prevent oxidative stress [14]. A cellular antioxidant activity assay could provide biologically relevant information on bioactive compounds in raw and processed food products. Shashirekha et al. [15] has comprehensively analyzed the phytochemical profiles of potato and their strong antioxidant activities which indicated the chemistry, biochemistry, and biological activities the identified major phytochemicals play in potato. Measuring antioxidant activity using biologically relevant assays gives strong supporting evidence in understanding the role of phytochemicals *in vivo* [16]. Carotenoid derivatives such as lutein, zeaxanthin, and violaxanthin are found in potatoes [17]. Brown [18] reported that the total carotenoid content of potatoes ranges from 35 *μ*g to 795 *μ*g per 100 g fresh weight and that dark-yellow cultivars contain approximately ten times more carotenoid than white cultivars. Compounds like anthocyanin, (a powerful antioxidant) chlorogenic acid are secondary metabolites that constitutes up to 80% of the total phenolic content of potato tuber [17].

Quercetin is a flavonoid found in large quantity in red and Russet potato cultivars that has demonstrated antioxidant and anti-inflammatory properties in *in vivo* and *in vitro* conditions [19]. Glycoalkaloids are produced in potato during germination to serve as an immune build-up mechanism by the tubers against pathogens, insects, parasites, and predators [20]. The primary glycoalkaloid in domestic potato is *α*-chaconin and *α*-solanine that is found in the outer layer of the potato skin [21]. Glycoalkaloid has also been reported by Friedman [20], to be linked with cholesterol, anti-inflammatory, antiallergic, and antipyretic effects.

## 4. Fungi Diseases of *Solanum tuberosum*

Pest and diseases are major constraints confronting potato farmers [22]. Postharvest diseases and food spoilage caused by fungal pathogens can occur during various stages of processing, such as harvesting, handling, storage, packaging, and transportation by both producer and the consumer [23]. At the postharvest, fungal rot disease pathogens are the main agents of rot diseases in fresh fruits and vegetables [24]. Numerous species of fungi are responsible for the cause of postharvest diseases [25]. It has also been reported by Moss [26] that developing countries within tropical regions, losses 50% of perishable crop plants to fungi pathogens. Sharma and Kulshrestha [27] proved also that *Colletotrichum* species of fungi causing disease can destroy 100% of stored fruits. By and large, fungal rot diseases of potato are very common in occurrence all over the World, as reported by Bongomin et al. [28]. Late blight diseases have constituted the most serious threat to potato farmer's productivity and value [29]. The resistance of new pathogenic strains of late blight disease has been noticed globally [29]. In the mid-1800s, history has revealed that the late blight disease devastated potato in Ireland specifically and Europe in general which led to a famine that claimed about one million lives [30]. Losses from late blight in developing countries are estimated to be about 10 million British pounds per annum [31]. Some important fungal diseases of potato are presented in Table 1.

## 5. Natural Resistance of Plant against Fungi

Natural resistance is the use of plants defense mechanisms in agricultural production to induce resistance against invading fungal pathogens [33]. Salicylic acid and its analogs are used to induce systematic acquired resistance in crops that are affected by diseases. Studies have shown that 30 g of benzo (123) thiadiazole-7-carbothioic acids-methyl ester (BTH) can protect the wheat crop against both *Puccinia recondita* and *Septoria* species for an entire season [34]. Jasmonic acid and its derivatives can induce resistance in crops affected with fungal diseases and production of compounds that have health benefits [35]. *Methyl jasmonates* supress post-harvest infections of strawberries by *B. cinerea* and reduces decay of marsh seedless grape fruit by *P. digitatum* in agriculture [25]. A study from Dorsaz et al. [36] identified other natural products such as Chitosan, B-aminobutyric acid, glucosinolates, propoliss fusapyrone, deoxyfusapyrone, ethephon, microbial products, and plant extracts that induce resistance against fungal pathogens. The products mentioned above are used globally to enhance quality and yields in agriculture [25, 37].

## 6. Artificial Fungicides in Crop Protection

Since 1800s, the use of fungicides has provided much needed relief in the management of plant fungal diseases in agricultural production. Although the use of fungicide yielded great benefits, the attendant risks to human health, wildlife, and the environment cannot be overlooked [38]. Fungicides are frequently toxic to nontargeted organisms such as earthworms, microbes, and humans causing ecosystem imbalance [33, 38, 39]. Fungicides are chemical agents used in the management of pathogens of plants. Specifically, some of the chemical components of the artificial fungicides are nonbiodegradable. Hence, they could pollute the water system in the environment such as river, lake, fish pond, and well [40]. Pesticides residue concentration in potato depends on numerous applications in the agricultural field, which can result to increase in pesticides residue consumption [41]. The study further showed that only chlorpyrifos pesticides residue was found in the tuber; however, none of the samples exceeded the permissible pesticides limit. Report from Randhawa et al. [42] and Lopez-Perez et al. [43] also showed similar result trends from *in vivo* pesticides application. An *in vivo* pesticides residue studies has shown much variation within species and genera of crops [44]. Due to the ignorance of farmers about the right application dosage in specific pesticides applications, lack of awareness on the right time intervals between applications and harvest as well as careless adherance to proper guardiance on pesticides application precautive measures were found to contribute to pesticides residue build up in potato. By and large all the side effect on the use of artificial pesticides can be averted, if the use of biopesticides are fully adopted by the farmers.

## 7. Biofungicides in Crop Production

Control of pathogenic fungi through induction of plant defences has prevented many people of the World from dying as a result of starvation or illness related to malnutrition or from dreadful epidemics outbreak that could occur from time to time [45]. To overcome the abovementioned consequences, caused by fungal disease pathogens, the use of various artificial fungicides is important. A recent study has shown that some of these agrochemicals or artificial fungicides are toxic to humans with negative impact on the environment, soil microorganisms, insects, and plant pollinators [38]. However the use of botanicals is attracting much attention from farmers, environmentalist, and consumers. The use of extracts obtained from numerous plants has recently gained popularity and scientific interest due to their antifungal activities [46–48]. The research works of Amadioha and Obi [49], Amadioha [50], and Okigbo [48] showed an inhibitory activities of some plants extracts the growth of mycelia of fungi pathogen. In addition to the aforementioned environmental hazards, there are also high chances of development of multiresistance strains of fungi pathogens [51]. Scientist and medical experts have shown increased interest in herbal medicine as they can be used to recognise the true health benefits of these herbs. Plants are one of the most important sources of medicine [52]. The medicinal plants are rich in secondary metabolites which are potential sources of drugs oil of therapeutic relevance. The advantages claimed for the therapeutic uses of medicinal plants in various disease cure are linked to their safety, economic and easy availability. Medicinal plants represent a rich source of antimicrobial agents [53]. Plants generally, produce many secondary metabolites which constitute an important source of fungicides, pesticides, and many pharmaceutical drugs.

The antimicrobial properties of *Azadirachta indica* leaves revealed high antifungal activity against *Pestalotiopsis thea* (tea plant). Also, *Allium sativum* bulbs showed maximum inhibitory potentials of spores germination of fungal pathogens [54]. Some active components of *Murraya koenigii* were reported to possess antifungal activity [55]. Reports by Ejechi et al. [56] and Ejechi and Ilondu [57] showed that herbs and other plants materials possess antifungal properties. Furthermore, Akinyosoye and Oladunmoye [58], reported that the antifungal efficacy of leaf and stem extracts of *Mirabilis jajapain* reduces the mycelial growth of four different strains of fungi pathogen. According to Ebele [59], the aqueous plant leaf extracts from *Carica papaya*, *Chomolaena odorata,* and *Acalypha cilia* were used to test the growth of the pathogenic fungi rot of *Carica papaya* fruits using 10, 20, and 30% concentration which significantly reduced the mycelial growth of *Aspergillus niger*, *in vitro* by 20.83%, *Botryodiplodia theobromae* 31.71%, and *Fusarium solani* 39.02%, respectively. Antimicrobial effects of *Vernonia amygdalina* and *Tridax procumbens* on fungi pathogens isolated from infected tomato fruit using water and ethanol solvents of 60–80% water and 20–30% ethanol concentration revealed that all the test plant extracts significantly reduces mycelia growth of the isolated pathogens. Higher concentration of both aqueous and ethanol favored higher mycelia growth reduction [60, 61]. Tables 2 and 3 show a summary of some of the selected biopesticides, while Table 4 shows the mechanisms of actions of phytochemicals on fungal pathogens that help in boosting agricultural productivity and consequently ensure food security.

## 8. Patterns of Biofungicides Usage

Biofungicides are used globally for the control of insects pests and diseases. The high patronage of residue-free crops is becoming higher due to the increasing health consciousness in India [66]. Abbey et al. [5] also reported that the demand for biofungicides will continue to increase globally. In 2011, North America dominated the global fungicides markets which accounted for about 40% of the global biofungicides demand [67]. The United States of America biofungicides market is valued at about 205 million American dollars which is anticipated to increase to approximately 300 million American dollars by 2020. In another development, European market for biofungicides is estimated at 200 million US dollars due to the stringent pestcide regulation and increasing demand for organic food. Kumer [67], reported that data on microbial biofungicides agents from agriculture and agric-food Canada and the United states of America environmental protection agency (EPA) indicate that more than 200 products are sold in the United States of America compared to 60 in Europe. The world market recorded that the cost for biofungicides patronage in 2011 was 1.3 billion American dollars, and it was anticipated to rise upto 3.2 billion American dollars by 2017 [67]. India has documented about 12 registered biofungicides under the Insecticides Act, 1968 (Table 5).

## 9. Mechanisms of Action of Agricultural Biofungicides

The mechanisms of action of biofungicides are broad in nature but are grouped as follows:

1. By competing with pathogens for nutrients with the rhizosphere
2. By directly killing plant pathogens
3. By indirect killing using secreted bioactive molecules against the plants pathogens
4. By Enhancing the plants cellular metabolic rates toward resisting pathogens
5. By enhancing active plants' growth beyond pathogens' attack [68].

### 9.1. Competition with Pathogens for Nutrients with the Rhizosphere

One of the key mechanisms of action of biofungicide application is by competing with pathogens for nutrients within the rhizosphere. The rhizosphere is a very nutritional stable environment and attracts diverse micro- and macro-organisms. These organisms utilize the exudates as source of nutrient. Pathogens of plants are the major users of these nutrients. However, the biofungicides immediately mop up the available nutrients in the rhizosphere, making the environment nutritional deficient for the pathogens to survive [68]. In this way, the presence of biofungicides encourages the growth of the plants.

### 9.2. Direct Attack by Killing Plant Pathogens

Studies have shown that biofungicides directly attack and kill the pathogens within and around the host: plant [69, 70]. The biofungicide acts as parasites of the pathogens which are within and on the plants surfaces. They used their special hyphae to attach, penetrate, and biodegrade the pathogens with their extracellular enzymes, thereby controlling the population of the plants pathogens and ultimately enhancing their growths [70].

### 9.3. Indirect Attack Using Secreted Bioactive Molecules against the Plants Pathogens

The active microbes which make up the biofungicide also destroy their target plants pathogens by secreting antimicrobial molecules which ultimately inhibits the growth of the pathogens [70, 71]. Some of the compounds acts like antibiotics and are effective against a score of pathogens of tubers and underground crops among others [70].

### 9.4. Enhancing the Plants Cellular Metabolic Rates towards Resisting Pathogens

Some of the studied biofungicides have been found to induce physiological and structural changes within the cells of the plants, thereby making them resist most parasitic attacks [72]. The presence of these biofungicides, activates some structural and chemcial changes in and on the plants which makes them less susceptible to a wide range of microbial attacks [70]. They also make the plants to respond actively against the intrusion of pathogens. Their effects could be systematic or local [70].

### 9.5. Enhanced Active Plants Growth beyond Pathogens' Attack

Certain biofungicides are made of cells that act as growth enhancers [70]. Such biofungicides stimulate the roots and the shoots of the plants to grow actively beyond the reach of their parasites [71]. Such plants can survive pest and disease attack, because the growth promoting microbes colonizing their tissues constantly replaces the worn out tissues and make the hot plants to resist further pathogens attack. They also enhance the growth of the plants by stimulating active absorptions growth that promotes mineral nutrients by the plants [70].

## 10. Agricultural Impact of Biofungicides on Potato Productivity

Potato is an indispensable cash crop and a carbohydrate-rich food crop in Africa and globally. Potato late blight disease, caused by *Phytophthora infestans*, is a global threat to potato farmers all over the World [29]. Late blight disease seriously affects the yields of potato and the application of biofungicides could ultimately boost their outputs and commercial values. Efficient control of late blight of potato has been observed using bacterial biocontrol agents in greenhouses or even in field experiments [73, 74]. A collection of pseudomonas isolated from the rhizophere and phyllosphere of potato was proved to show *in vitro* protective effects against *Phytophthora infestans* [75]. Several studies have suggested ways to enhance the yield of this all important crop [76]. A study revealed that a calculated amount of fertilizers and fungicides can significantly reduce the incidence and severity of late blight in potatoes [77]. The biofungicide Taegro (*Bacillus subtilis* var. *Amyloliquefaciens* strain FZB24) has been reported to demonstrate effective potential in decreasing the incidence and severity of late blight disease in potato [78]. A dynamic participation of the private sector in seed production and integrated pest and disease management (IPDM) has been identified as an effective approach towards maintaining a sustainable potato production and food security in Rwanda [76, 79]. Control of pests and diseases with botanicals, which are ecofriendly, cannot be overemphasized. According to Kumar and Singh [80], exploitation of biofungicide remains an indefensible agent for agricultural produce considering the increasing demands for organic foods. In organic farming, copper is used despite its persistence in soil and its organism toxicity. To replace copper, suspension of ground powder from three botanicals such as bark of *Frangula alnus* (Buckthorn), roots of *Rhem palmatum* (Medicina rhubarb), and the gall of the *Galla chinensis* (nut gall tree.) were tested by multiyear field experiment [81]. The result revealed that botanicals could replace copper under field trial conditions and *Fragula alnus* reduces late blight leave occurrences [81].

Three biofungicides called Biocont-T, Fungus stop, and Polyversum with *Trichoderma harzianum*, citric acid with mint oil and *Pythium oligandrum* as the active ingredients, respectively, were tested against *Fusarium oxysporium*, a soil born fungal disease that causes potato vascular wilt diseases [82]. The result revealed that fungus stop proved to be most effective by inhibiting about 72–76% mycelial growth of the pathogen on potato dextrose agar (PDA) media after six days incubation at 25°C. followed by Bicont-T with mycelial growth inhibition of 37–67% and lastly, polyversum with little inhibitory impart [82].

Methanoic extracts of six plant leaves, namely, *Lavandula officinalis*, *Eucalyptus camaldulensis*, *Artemisia annua*, *Tymus vulgaris*, *Satureja mutica*, and *Datura stramonium,* were examined and proved to exhibit significant antifungal activity against *Fusarium solani*, the causal agent of potato rot disease in *in vitro* and *in vivo* trials [83].

The *in vitro* evidence of ethanolic extracts of *A. indica* seeds in reducing potato tuber infestation and damage by potato tuber moth (PTM) in stored potatoes in Ethiopia has also been identified [84]. In the same vein, the aqueous leaf extract of lemon grass leaf reduces mycelial growth and spore germination in *P. infestans* and *A. solani* [85]. Essential oil of *C. citratus* has also been reported to inhibit the myclial growth of *P. infestans* in the laboratory [86].

*Lantana camara* is an evergreen, hardy shrub that originates from the American tropics. *Lantana* has been reported to reduce the mycelial growth of late blight in potato under *in vitro* condition [87]. Extracts of *Lantana* leaves and flowers were reported to reduce the rate of *in vitro* sporangia germination and mycelial growth of *P. infestans* and *A. solani* in potato by 50%, consequently leading to a 37% increase in potato yield [85].

Another experiment conducted in potato stores has proved that *L. camara* leaf powder application on potato tubers at two month-interval, at a rate of 50 g per bed (2 m by 3 m), reduced potato infestation and damage by potato tuber moth (PTM) [84].

In another findings on, *in vivo* trial using biochar produced from *L. camara*, *Ipomoea cornea* var. Fistolosa, and rice husk, showed that potato yield significantly improved [88].

## 11. Future Perspective Usage of Biopesticides

To overcome the challenges confronting farmers through the use of chemical pesticides, the use of botanicals has been the main focus of plant protection research. In Africa, plant extracts from *A. sativum*, *A. indica*, *C. citratus*, *D. stramonium*, *L. camara,* and *Ocimum gratissimum* are the most commonly studied, for potato pathogens and pests. The recent use of botanicals in potato research in Africa is quite limited [89].

Furthermore, it has often been observed that botanically-based studies are often restricted to laboratory settings, with less attention focusing on field data findings [89]. Future research should be directed to effective formulation, concentration, and the subsequent effect on nontarget organisms in the field. In addition, integrating botanicals with other control measures could improve biopesticide efficacy.

## 12. Conclusion

Potato cultivation and production has been gaining ground globally due to its spotted role in alleviating food insecurity despite the constraint of food losses incurred at the pre- and post-harvest stages of production from fungal diseases. Reliance on the use of artificial fungicides has become unpopular due to the environmental hazard and toxicity effect which has refocused the attention of farmers towards the use of biofungicides in the control of pests and diseases involved in the reduction of food crops generally and potato production in particular. The monitoring trends of the fast adoption of the use of biofungicides all over the world has revealed the consciousness of health risks involved with the use of artificial fungicides. As a result, government policy makers should encourage the use of biofungicides by funding research, given publicity through sponsoring conferences in collaboration with the United Nations Organization and their stakeholders, to enhance biopesticide production research funding.

## Figures and Tables

**Table 1:** Important fungal disease of potato.

Disease name	Causal organism
Late blight	*Phytophthora infestans*
Early blight	*Alternaria solani*
Wart disease	*Synchytrium endobioticum*
Stem canker and black scurf	*Rhizoctonia solani*
Powdery scab	*Spongospora subterrannea*
Pink rot	*Phytophthora erythroseptica*
Silver scurf	*Helminthosporium solani*
Watery wound rot	*Pythium ultimum* and *P. debaryamum*
Gangerene	*Phoma exigua*
Dry rot	*F. coeruleum*, *F. eumartii*, *F. oxysporum,* and *F. sulphureum*
Skin spot	*Polysecytalum pustulans*
Wilting	*Verticillium alboatrum*
Charcoal rot	*Macrophomina phaseolina*

Source: [32].

**Table 2:** Important plant families having plants with antifungal activity.

Family	Number of plants
*Asteraceae*	39
*Euphorbiaceae*	63
*Fabiaceae*	57
*Leguminosae*	60
*Meliaceae*	>500
*Myrtaceae*	72
*Ranunculaceae*	55
*Rosaceae*	27
*Rutaceae*	39

Source: [62, 63].

**Table 3:** Botanicals with antimicrobial activities.

Common name	Scientific name	Compound	Class	Activity
Apple	*Malus pumila* mill	Phloretin	Flavonoid	General
Ashwagandha	*Withania somnifera*	Withafarin A	Lactone	Fungi
Bael tree	*Aegle marmelos*	Essential oil	Terpenoid	Fungi
Blue germ tree	*Eucalyptus globules*	Tannin	Polyphenol	Fungi, bacteria
Onions	*Allium cepa*	Alicin	Sulfoxide	Fungi, bacteria
Thyme	*Thymus vulgaris*	Caffeic acid	Terpenoids	Fungi, bacteria
Turmeric	*Curcuma longa*	Curcumin	Terpenoids	Fungi, protozoa
Thome apple	*Datura sramonium*	Hyoscymine	Scopolamine	Fungi
Castor	*Ricinus communis*	Rcinine	Alkaloid	Fungi
Neem tree	*Azadirachtaindica*	Azadirachtin	Terpenoids	Fungi, bacteria
Garlic	*Allium sativum*	Alicin	Solfoxide	Fungi, bacteria
Black pepper	*Piper nigrum*	Piperine	Alkaloid	Fungi

Source: [64].

**Table 4:** Mechanisms of action of phytochemicals on fungal pathogens.

Name of the compound	Mode of action
Simple phenols	Membrane disruption and substrate deprivation
Phenolic acids	Bind to adhesins, complex with cell wall, inactivate enzymes
Terpenoids	Membrane disruption
Essential oils	Membrane disruption
Alkaloids	Intercalate into cell wall
Tannins	Bind to proteins, enzyme inhibition, substrate deprivation
Flavonoids	Bind to adhesins, complex with cell wall, inactivate enzymes
Coumarins	Interact with eukaroyotic DNA
Lectins	Form disulfide bridges
Polypeptides	Form disulfide bridges

Source: [65].

**Table 5:** Biopesticides registered under the Insecticides Act, 1968.

S/N	Name of biofungicides
1	*Bacillus thuringiensis* var isrealensis
2	*Bacillus thuringiensis* var kurstaki
3	*Bacillus thuringiensis* var gallerine
4	*Bacllus sphaericus*
5	*Trichoderma viride*
6	*Trichoderma harzianum*
7	*Pseudomonas fluoresens*
8	*Beauveria bassiana*
9	NPV of *Helicoverpa armigera*
10	NPV of *Spodoptera litura*
11	Neem-based pesticides
12	Cymbopogon

Source: [66].

## Data Availability

The data used to support the findings of this study can be obtained from the corresponding author upon request.
